# The molecular mechanism of human stem cell-derived extracellular vesicles in retinal repair and regeneration

**DOI:** 10.1186/s13287-023-03319-2

**Published:** 2023-04-12

**Authors:** Mei Yang, Guang-hua Peng

**Affiliations:** 1 Laboratory of Visual Cell Differentiation and Regulation, Basic Medical College, Zhengzhou University, Zhengzhou, 450001 China; 2 Department of Pathophysiology, Basic Medical College, Zhengzhou University, Zhengzhou, 450001 China

**Keywords:** Retina, Repair, Regeneration, Stem cell, Extracellular vesicle, Therapy

## Abstract

Extracellular vesicles (EVs), including microvesicles (MVs) and exosomes, play a critical role in metabolic regulation and intracellular communication. Stem cell-derived EVs are considered to have the potential for regeneration, like stem cells, while simultaneously avoiding the risk of immune rejection or tumour formation. The therapeutic effect of stem cell-derived EVs has been proven in many diseases. However, the molecular mechanism of stem cell-derived EVs in retinal repair and regeneration has not been fully clarified. In this review, we described the biological characteristics of stem cell-derived EVs, summarized the current research on stem cell-derived EV treatment in retinal repair and regeneration, and discussed the potential and challenges of stem cell-derived EVs in translational medicine.

## Background

The retina is the only nerve tissue in the body that senses and transduces light signals and has a delicate anatomical structure and complex physiological environment. The core cells that sense and transduce light signals are photoreceptors and retinal ganglion cells (RGCs). The retinal pigment epithelium (RPE) sits between the choroid and photoreceptors, transmitting nutrients to photoreceptors and RGCs. In the retina, cells that perform immune-like functions include microglia, astrocytes, and Müller cells [1]. Astrocytes and Müller cells are involved in forming the blood‒retinal barrier (BRB) [1], while microglia, when activated, can phagocytose cellular debris, performing a macrophage-like function [2]. The retina is one of the body's most energy- and oxygen-intensive tissues [3]. Rapid energy consumption generates an abundance of reactive oxygen species (ROS), creating a localized, highly oxidative microenvironment [4]. In a healthy eye, a large quantity of antioxidants depletes ROS and prevents them from damaging retinal cells [5]. Here, the oxidative and antioxidant reactions are in balance. However, imbalances in oxidation and antioxidation caused by various factors, such as genetic mutations, ageing, and poor lifestyle, can lead to decreased production of reducing agents and a considerable accumulation of ROS [6]. Unreduced ROS attack retinal cells, creating a pathological environment in the eye. Age-related macular degeneration (AMD), diabetic retinopathy (DR), and glaucoma are eye diseases that can lead to vision loss. ROS are induced in these diseases, leading to retinal degeneration [6]. Moreover, inflammation is another common trigger for retinal degeneration. Proinflammatory factors in the retina are mainly produced and released by activated microglia, predominantly tumour necrosis factor-α (TNF-α) and interleukin (IL)-1β, as well as IL-3, IL-6, IL-8, IL-10, IL-12, and IL-18 [1, 7]. Optic ganglion cells are impaired, and their number decreases. The retina becomes weak and thin, the ability to sense light is impaired, and the patient suffers varying degrees of visual loss [8]. Activation of the inflammatory response can be observed in almost all retinal diseases [9, 10]. In addition to oxidative stress and the inflammatory response, multiple damage mechanisms, such as abnormal metabolism and disturbances in neovascular growth, shape the abnormal intraocular environment in the pathological state [11, 12]. Each factor can occur either individually or simultaneously and form vicious feedback cycle.

Traditional treatments for retinal degeneration or damage include surgery and medications, such as eye removal, anti-inflammatory drugs, and immunosuppressive drugs [13]. However, conventional treatments are unable to replace and replenish dead retinal cells or restore the patient's ability to see. For this reason, new retinal repair therapies need to be developed. Two new therapies are already in clinical trials and may be effective in repairing retinal damage: gene therapy and cell transplantation [14, 15]. Gene therapy uses an artificially modified adeno-associated virus (AAV) vector to insert a target gene into a target cell to replace the mutated gene and resume normal cellular function. For example, adeno-associated virus vector serotype 2 (AAV2) targets the RPE to treat Leber congenital amaurosis and other retinal dystrophies caused by recessive mutations in the RPE65 gene [16]. Cell transplantation therapy replaces degenerated cells by replenishing the lesion with normal retinal cells or stem cells to reconstruct neural signalling pathways and a healthy retinal microenvironment [15]. In particular, advances in the cultivation of induced pluripotent stem cells (iPSCs) mean a theoretically unlimited supply of material for cell transplantation therapy [17]. However, there are still some substantial drawbacks to both therapeutic approaches. Studies in which AAV-mediated retinal gene therapy was applied have independently reported intraocular inflammation [18]. In some cases, long-term test results showed a gradual loss of efficacy after an initial improvement in visual function. This may be due to the activation of innate pattern recognition receptors (PRRs), such as toll-like receptor (TLR)-2 and TLR-9, by AAV, leading to the release of inflammatory cytokines and type I interferons [14]. The clearance of AAV by the body limits the effectiveness of long-term treatment. In addition, AAV needs to be injected into the subretinal space in the lesion area to achieve an optimal therapeutic effect, which requires a high level of skill on the part of the physician [19]. For cell therapy, the survival rate of the implanted cells has a significant impact on the outcome of the treatment. Experimental results on RPE transplants have shown that cells injected as a suspension do not consistently form an RPE monolayer at the time of transplantation and have a low long-term survival rate [15]. In a pathological environment of oxidative stress and an inflammatory response, it is difficult to ensure that the implanted cells reach the required number of viable cells [20]. When transplanting stem cells, the possibility of differentiation into unexpected cell types or even cancer must be addressed. All of these issues challenge existing therapeutic approaches.

Recently, a new therapeutic approach has begun to be tested in retinal damage repair experiments. Extracellular vesicles (EVs), tiny particles secreted by various cells, have been shown to play a wide range of roles in the physiological regulatory processes of the body and various diseases [21]. Among these, EVs derived from stem cells, particularly mesenchymal stem cells (MSCs), have been shown to promote retinal damage repair and restore visual ability [22, 23]. The reparative effects of stem cell-derived EVs have been demonstrated in many retinal diseases, including AMD, diabetic DR, laser- or drug-induced retinal degeneration, glaucoma, autoimmune uveitis, and damage to optic ganglion cells and their axons [22], and have been shown to inhibit inflammatory responses, offer neuroprotection, improve retinal ischaemia, and protect against photoreceptor apoptosis, showing good therapeutic potential. Following gene therapy and cell replacement therapy, stem cell-derived EVs may become a new therapeutic approach for retinal diseases.

## Main text

### Introduction to extracellular vesicles

The first electron microscopy images of nanosized particles described as ‘platelet dust’ were published by Peter Wolfe in 1967 and provided the earliest designation for EVs [24, 25]. Until the 1990s, the prevailing view, represented by Rose Johnstone, described EVs as a means for cells to selectively eliminate metabolic waste products [25, 26]. However, as more findings have emerged in the twenty-first century, researchers have found that EVs possess a greater range of functions. EVs have now been identified as a new mode of intercellular communication, mediating various biological processes, including neural signalling, regulating immune function and inflammation, and mediating tumour growth and distant metastasis [27–29].

According to a position statement issued by the International Society for Extracellular Vesicles (ISEV) organization in 2018, EVs are generally defined as particles naturally released from various cells that are bounded by a lipid bilayer and unable to replicate [30]. EVs are typically classified into three subtypes based on differences in size and biogenesis processes: exosomes, microvesicles (MVs), and apoptotic vesicles [31, 32]. Exosomes range from approximately 30–150 nm in diameter, and their formation and release follow a specific biological process known as the endosomal pathway, which distinguishes exosomes from other EVs. The general process of the endosomal pathway can be summarized as a double folding of the cytoplasmic membrane. First, the cytoplasmic membrane invaginates to form the endosome. The endosome membrane then folds back on itself again to form multivesicular bodies (MVBs), within which intraluminal vesicles (ILVs) are formed. Mature MVBs are transported to the plasma membrane, and after fusion with the membrane they release the ILVs, now known as exosomes, outside the cell [33, 34]. Moreover, endosomal sorting complexes required for transport (ESCRT) proteins are involved in the formation of ILVs in MVBs and the sorting of cargo [35]. ESCRT proteins represent a family of proteins that are found in the ESCRT complex and have three functions: they recognize ubiquitinated cargo and prevent its degradation; then, they deform the membrane and sort cargo in or out; and finally, they form ILVs that wrap around the sorted cargo [36]. The ESCRT complex comprises four subcomplexes, namely, ESCRT-0, ESCRT-I, ESCRT-II, and ESCRT-III and the associated AAA ATPase Vps4 complex, which recognizes protein cargoes and sorts them into ILVs [37]. In addition to relying on the ESCRT pathway, exosome biogenesis can also occur through mechanisms that are not dependent on the ESCRT pathway [32]. These two pathways may not be completely independent and can coordinate with each other, but different subpopulations of exosomes may utilize different mechanisms [38].

MVs, which range in diameter from approximately 100–500 nm, are produced by direct shedding from the cytoplasmic membrane of living cells; this is called cytoplasmic ‘budding’. This process involves remodelling the actin cytoskeleton and disrupting local lipid bilayer asymmetry [39]. More specifically, the actin cytoskeleton undergoes proteolysis due to increased calcium ion concentrations [40]. Phosphatidylserine ectoplasmosis causes a change in lipid distribution, with MVs tending to detach from the surface of their source cell, and the enzymes scramblase, floppase, and flippase, which ‘flip’ different phospholipids in and out of the plasma membrane, are involved in this process.

Apoptotic vesicles are larger than 500 nm in diameter, and their biogenesis is similar to that of MVs, except they are released by dying apoptotic cells. There is evidence that apoptotic vesicles produced during apoptosis may play an important immunomodulatory role rather than simply packing away the ‘remains’ of the cells [41]. Additional research on apoptotic vesicles may lead to future breakthroughs in therapeutic strategies using EVs.

The EVs discussed in this paper mainly cover exosomes and MVs produced by living cells. Following the recommendations of the ISEV position statement, exosomes refer specifically to EVs produced and released via the endosomal pathway, while MVs refer specifically to plasma membrane-derived EVs [30].

The composition of exosomes varies depending on the cellular origin, but there are still components found in most exosomes, including endosome-associated proteins such as Rab GTPase, SNAREs (soluble N-ethylmaleimide-sensitive factor attachment protein receptors), annexins and flotillin, MVB production-associated proteins such as Alix and Tsg101, a family of four transmembrane proteins such as CD63, CD81, and CD9, heat shock proteins HSP70/90, and major histocompatibility class I (MHC I) antigens [42, 43]. In addition, in contrast to the plasma membrane, exosomal membranes are highly enriched in cholesterol, sphingomyelin, and hexosylceramide rather than phosphatidylcholine and phosphatidylethanolamine [39]. In contrast, MVs express CD40, selectins, integrins, and cytoskeletal proteins, and their membranes are highly enriched in cholesterol, phosphatidylserine, and diacylglycerol [39].

**Fig. 1:**
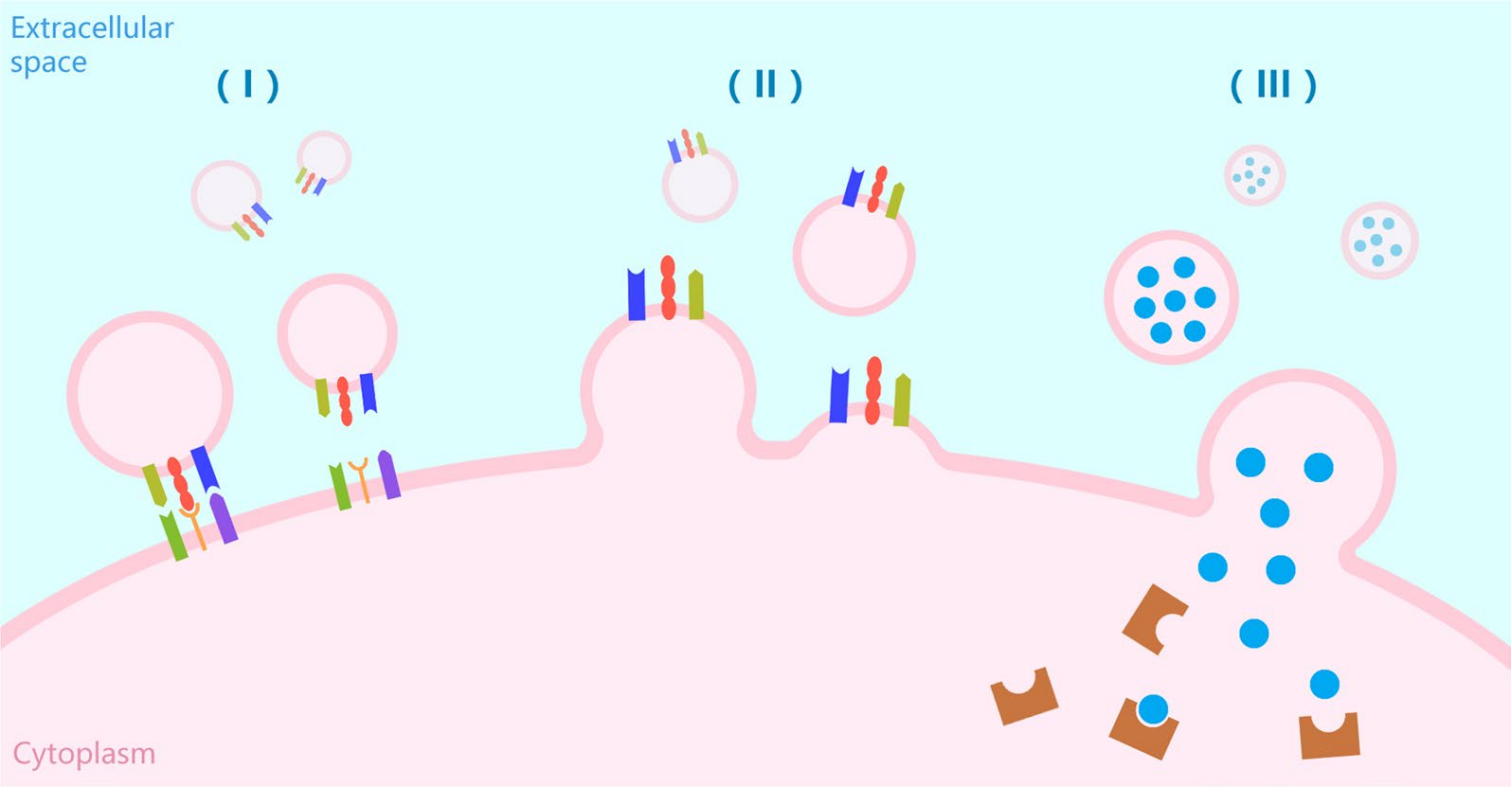
The signalling delivery methods of extracellular vesicles. (I) EVs carry specific signalling molecules on the surface of their lipid membranes and bind to receptors on the surface of target cells; (II) EVs transport receptors via membrane fusion with recipient cells; (III) EVs release the cargo packed inside the vesicle directly into the recipient cytoplasm. This image was drawn by the first author

As players in intercellular communication, EVs can carry specific cargoes, including various proteins, lipids, DNA, mRNA, micro-RNA (miRNA), other small noncoding RNAs (ncRNAs), and a variety of bioactive substances. The cargo-carrying process is precisely regulated by intracellular cargo-sorting mechanisms. The sorting and loading of cargo from exosomes are mainly accomplished through ESCRT-dependent and ESCRT-independent pathways [21, 44, 45]. The cargo species and cargo-sorting mechanisms of MVs remain to be studied further, but the small GTP-binding protein ARF6-regulated recycling pathway may play an important role in guiding cargo selection [46–48]. An interesting phenomenon is the similarity between the active components carried by MVs and those of the parent cells and the presence of some active analogues and source cell-specific markers [49]. Recent studies have shown that EVs can participate in intercellular communication in multiple ways. EVs carry specific signalling molecules on the surface of their lipid membranes, which act as a ‘signalling platform' to bind to receptors on the surface of target cells. EVs remain relatively independent and intact in this method. EVs can also transfer their surface-encapsulated receptor molecules via membrane fusion with recipient cells. EVs can fuse with the recipient cell and transfer their surface-encapsulated receptor molecules to the target cell membrane, altering the receptor surface pattern of the recipient cells or releasing the cargo packed inside the vesicle directly into the recipient cytoplasm to exert regulatory effects [42, 43]. The signalling delivery methods mentioned above are shown in Fig. 1.

Given their comprehensive biological functions and ability to shuttle macromolecules between cells, EVs provide a unique platform for the development of a new class of therapeutic approaches. Several experimental studies have reported that EVs secreted by stem cells mimic the immunomodulatory functions and regenerative capacity of stem cells and play an integral role in the repair of damaged tissues or organs by stem cells [50–52].

### The role of stem cell-derived EVs in retinal damage repair

The repairing effect of stem cells on the retina is partly mediated by the EVs they release. Inhibition of endosomal-derived EV production by MSCs using a specific GW4869 inhibitor revealed that the therapeutic effect of MSCs on retinal damage was greatly affected [53, 54]. Overall, stem cell-derived EVs promote cell proliferation, resist apoptosis, suppress inflammatory responses, repair retinal structures, and restore visual capacity in various retinal disease models. Most studies on retinal damage repair by stem cell-derived EVs have shown that miRNAs mainly mediate the molecular mechanisms underlying the actions of stem cell-derived EVs [55, 56]. Ben Mead et al. knocked down Argonaute-2, a key miRNA effector molecule, in bone marrow mesenchymal stem cells (BMSCs) and implanted BMSC-derived EVs (BMSC-EVs) into the eyes of optic nerve crush model mice [57]. They noted that the knockdown of Argonaute-2 impaired the ability of BMSC-EVs to promote the survival of RGCs and axon regeneration compared to controls, suggesting that miRNAs play a critical role in the regulation of physiological activity in target cells by EVs. miRNAs within EVs are delivered to receptor cells and activate or repress target genes by regulating their expression in receptor cells. These downstream signalling pathways are mainly involved in cell proliferation and PTEN inflammatory responses. In a model of diabetic retinopathy, human adipose mesenchymal stem cell (hADSC)-derived EVs negatively regulated integrin subunit α1 (ITGA1) expression in target cells via miR-192 [58]. Besides, human umbilical cord MSC (hucMSC)-derived EVs were loaded with miR-126, miR-17-3p, and miR-27b. miR-126 targeted high-mobility group box 1 (HMGB1) and inhibited the expression of the NLRP3 (NOD-like receptor family, pyrin domain-containing) inflammasome, NF-κB (nuclear factor kappa B)/P65, VCAM-1 (vascular cell adhesion molecule 1), and ICAM-1 (intercellular adhesion molecule 1) [59]. Moreover, miR-17-3p targeted signal transducer and activator of transcription 1 (STAT1), reduced inflammatory factor expression and ROS content, increased the activity of the antioxidants superoxide dismutase (SOD) and glutathione peroxidase (GSH-Px), and attenuated oxidative damage and inflammatory responses to the retina [60]. miR-27b inhibited transforming growth factor beta (TGF-β)-induced epithelial–mesenchymal transition by targeting homeobox C6 (HOXC6) and attenuated subretinal fibrosis [61]. Additionally, Chun-Lei Deng et al. inhibited TGF-β-induced subretinal fibrosis by targeting *N*-methyl-*N*-nitrosourea (MNU)-induced photoreceptor loss in mouse models transplanted with mouse BMSC-EVs and found that miR-21 maintained photoreceptor survival by targeting programmed cell death 4 (PDCD4) to inhibit apoptosis [53].

Stem cell-derived EVs have unquestionable neuroprotective effects on the retina. Stem cell-derived EVs promote the survival and proliferation of photoreceptors and RGCs and inhibit apoptosis, as observed in retinal ischaemia models [62, 63], glaucoma models [64], diabetic retinal degeneration models [58], and optic nerve crush models [65]. Yi Cui et al. found that rat BMSC-EVs upregulated the Bcl-2/Bax ratio, downregulated caspase-3 activity, stimulated the phosphorylation of the serine/threonine kinase Akt, and activated the PI3K/Akt signalling pathway to promote the proliferation of RGCs and inhibit apoptosis [65]. However, the regenerative effects of stem cell-derived EVs on axons are controversial. Two separate studies by Dongyan Pan et al. and Seyedeh-Zahra Seyedrazizadeh et al. were carried out; both established animal models of optic nerve crush [66, 67]. Both studies showed that MSC-EVs could promote the survival of Brn3a^+^ (brain specific homeobox/POU domain protein 3a-positive) RGCs. However, Pan et al. did not observe an increase in GAP43^+^ (growth-associated protein 43-positive) axon counts, which in contrast to Seyedrazizadeh et al. This discrepancy may be because the two studies used different species of experimental animals with MSCs from different tissue sources. The results of these two experiments suggest that EVs secreted by MSCs from different tissue sources may exhibit some degree of variability in their ability to repair tissues. Comparing different tissue-derived MSCs and selecting the appropriate species for EV extraction may be a direction for further research. In contrast of optic nerve injury, MSC-EVs have been shown to play a role in promoting axonal regeneration in other central nervous system (CNS) diseases [68, 69]. In an animal model of spinal cord injury, MSC-EVs enhanced axonal regeneration by targeting phosphatase and tensin homolog (PTEN) expression and inhibiting the PTEN-mTOR pathway [70]. This result suggests that MSC-EVs may promote axonal regeneration in RGCs in retinal diseases.

**Fig. 2:**
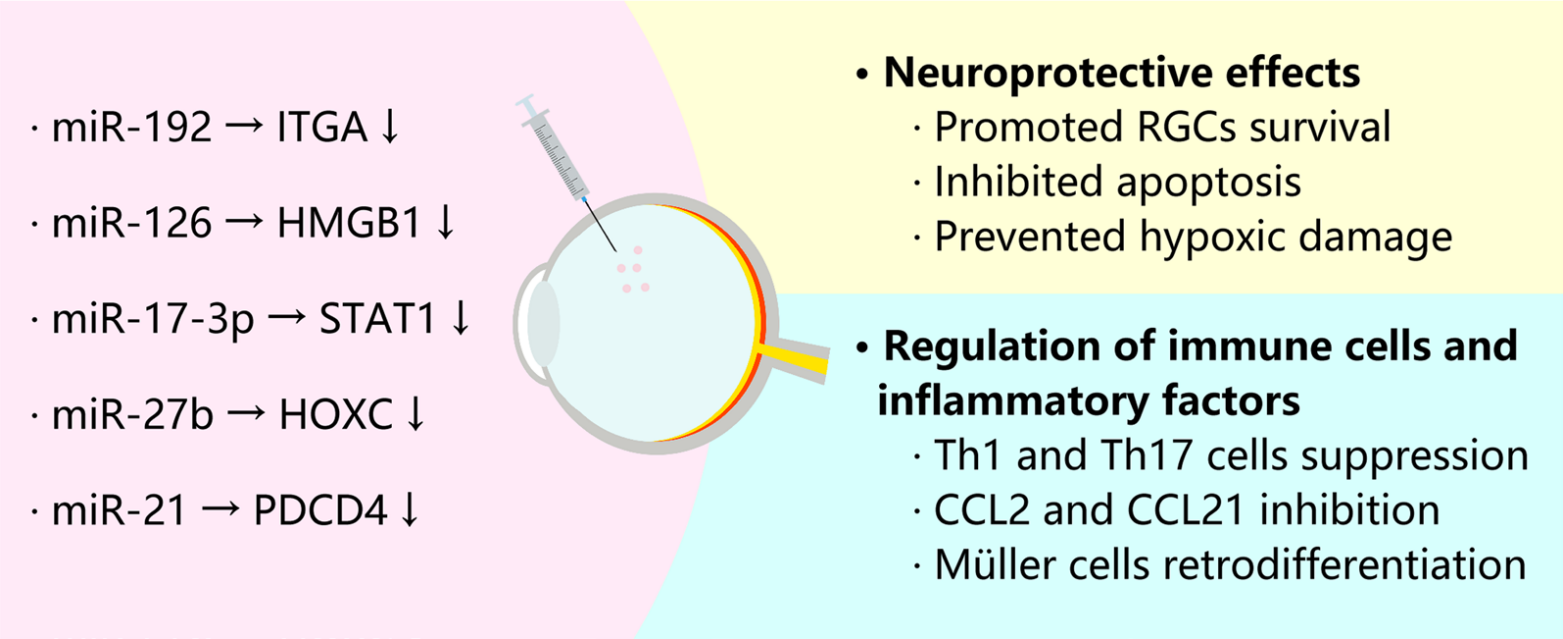
The role of stem cell-derived EVs in retinal damage repair. The left part shows the molecular mechanism of microRNAs and the right part shows the biological effects on retinal repair. ITGA1: integrin subunit α1; HMGB1: high-mobility group box 1; STAT1: signal transducer and activator of transcription 1; HOXC6: homeobox C6; PDCD4: programmed cell death 4

Another essential function of stem cell-derived EVs is the regulation of immune cells and inflammatory factors [71]. Human embryonic stem cell (hESC)-derived EVs increased brain-derived neurotrophic factor (BDNF) expression in Müller cells, activated the Wnt pathway, and contributed to Müller cell dedifferentiation [72]. hucMSC-EVs inhibited the chemical activation of immune cells by acting on C–C motif chemokine ligand 2 (CCL2) and CCL21 in autoimmune uveitis models. Additionally, they reduced the migration and infiltration abilities of T cells and other inflammatory cells into the eye [73, 74]. hucMSC-EVs also downregulated MCP-1 (also called CCL2) mRNA expression in retinal receptor cells in a laser-induced optic nerve injury mouse model, reducing damage to retinal cells from excessive inflammatory responses [75]. hBMSC-EVs inhibited the activation of antigen-presenting cells and suppressed the development of T helper 1 (Th1) and Th17 cells, preventing type 1 diabetes and the experimental autoimmune development of retinitis [76]. The role of stem cell-derived EVs in retinal damage repair is summarized in Fig. 2.

A special type of treatment based on BMSC-EVs, platelet-rich plasma (PRP), has also been found to play an active role in many disease models, including alopecia, acne scarring, and skin regeneration [77]. PRP is enriched in various growth factors, such as vascular endothelial growth factor (VEGF), platelet-derived growth factor (PDGF), epidermal growth factor (EGF), fibroblast growth factor (FGF), insulin-like growth factor 1 and 2 (IGF-1 and IGF-2), transforming growth factor-β1 (TGF-β1), hepatocyte growth factor, angiopoietin-1, cytokines, and plasma proteins [78]. Autologous PRP could promote the proliferation and reduce the apoptosis of RGCs in rabbit MNU-induced retinal degeneration model [79]. In the treatment of full-thickness macular holes (FTMHs), revisional pars plana vitrectomy with PRP resulted in a closure rate of 57.1–91% for refractory FTMHs [78]. However, additional experimental data have suggested that PRP may contribute to retinal degeneration. PRP is enriched in cytokines and other proinflammatory substances that increase the activity of microglia and Müller glial cells and promote inflammatory responses and phagocytosis [80, 81]. Moreover, PRP does not appear to exert a beneficial effect on the survival of RGCs, even in the absence of glial cells [81]. PRP-derived EVs induced ROS production and inhibited SOD activity in the eyes of mice with diabetic retinopathy, and they damaged the retinal endothelium by targeting the inflammatory response mechanism that is regulated by the TLR4 signalling pathway, in which CXCL10 plays a key role [82]. Overall, the therapeutic effect of PRP on the retina can be controversial.

### Advantages and challenges of clinical trials on stem cell-derived EVs for the repair of retinal damage

Research on the repair of retinal damage by stem cell-derived EVs has received increasing attention in recent years, but most experiments are still at the animal model stage. The only existing clinical trial was conducted and reported by Xiaomin Zhang et al. [83]. They used hucMSCs or hucMSC-EVs in seven patients with large and refractory macular holes. Each patient had the disease for at least one year. Two of the seven patients received an injection of 5 × 10^3^ hucMSCs and underwent tamponade procedures, and the remaining five received low (20 μg) or high (50 μg) doses of hucMSC-EVs intraocularly. EVs were extracted and isolated from the conditioned medium of hucMSCs according to the criteria for exosomes. One patient treated with hucMSCs developed a fibrous membrane on the retinal surface after 1 month. Moreover, a patient treated with a high dose of hucMSC-EVs developed moderate inflammation in the anterior chamber that disappeared three days after using steroid eye drops. The dose of EVs was reduced to 20 μg in this patient, and major inflammation was not observed again. All patients were followed up for 0.5–3 years, and during this time, no long-term side effects of hucMSCs or EVs were observed, and no evidence of major inflammation or risk of teratoma development was seen. All patients, including the two patients who were reinjected with hucMSCs or hucMSC-EVs after an adverse reaction, showed varying degrees of improvement in visual acuity and/or macular structure. This clinical trial confirms that hucMSC-EVs can successfully repair retinal damage and restore visual capacity and has great potential to be extended to MSC- or even other stem cell-derived EVs. Furthermore, the results showed that hucMSC-EVs tended to have a lower incidence of adverse effects than hucMSCs. However, this result could not be statistically proven due to the small sample size.

Some researchers have discussed the therapeutic role of stem cell-derived EVs, particularly MSC-EVs, in other diseases, pointing to their low immunogenicity as an advantage for transplantations [84]. In contrast to stem cell transplantation, EV transplantation does not seem to require strict adherence to homologous transplantation guidelines. Use of human cell-derived EVs in rodents can still achieve significant therapeutic results, and no severe immune rejection reactions occur. Stem cell-derived EVs, as a new therapeutic modality, can overcome some of the innate deficiencies of stem cell transplantation therapy. For example, studies in which MSC-EVs were used to treat liver cirrhosis suggested that EVs obtained by in vitro isolation can be free of cell viability issues and possess better organ permeability [85]. As a cell-free therapy, EVs also do not lead to a risk of stem cell malignancy. EVs are more tolerable of the complex pathological environment of the eye. Experimental results of studies in which the use of EVs for the treatment of CNS diseases was explored have shown that EVs can cross complex biological barrier systems such as the blood–brain barrier (BBB) and the BRB [31, 50]. Additionally, a prior study found that the treatment effect of the group in which an intravenous method of EV administration was used was significantly higher than that of the control group administered saline, demonstrating the possibility of transporting EVs to the site of the eye lesion through the peripheral circulation pathway, showing that EVs have some organ selectivity [86]. Finally, stability is also one of the advantages of EVs. In a suitable protective solution, EVs can be stored at − 80 °C for 6 months and retain their original biological function [87]. As a result, EV treatment is less costly than other applicable clinical treatments.

However, although several experiments have demonstrated the potential of stem cell-derived EVs in repairing ocular diseases and retinal damage, use of stem cell-derived EV therapies in the clinical setting still faces some challenges. The effect of EVs is transient compared to that of stem cell therapy because free EVs are quickly cleared after injection into the vitreous [88]. Immunofluorescence assay results showed that BMSC-EVs remain on the retinal surface of mice for approximately 4 weeks [63]. On the basis of glaucoma models, researchers have recommended that EVs be injected more than once per month, suggesting that EVs injected less frequently are less likely to maintain efficacy [22]. The duration of stem cell transplantation benefits, in contrast to that of EV treatment, applied to improve the visual function of patients ranged from months to years [89, 90]. This means that to be effective, treatments with EVs require longer cycles, more frequent applications, and larger doses of medication, resulting in low patient compliance, expensive treatment costs, and many other problems. Moreover, for EV translation into clinical use, additional practical difficulties need to be overcome. One issue that cannot be ignored is the potential for toxic doses of EVs, which becomes critical to explore in the experimental design phase. Zhang et al. admitted that they did not find information on toxic doses of EVs in retinal treatments prior to the start of their experiments [83]. In their results, there was indeed one case where an inflammatory reaction occurred at a high dose of hucMSC-EVs; however, the case improved when the dose was lowered. Although the available findings suggest a dose-dependent therapeutic effect of EV therapy [91], the findings of Zhang et al. suggest that high doses of EVs may increase the risk of an inflammatory response. Therefore, it is necessary to determine the toxic dose of EVs. Second, an additional challenge of EV therapy is the considerable heterogeneity of different EV subtypes. For example, Lopez-Verrilli et al. found that exosomes and MVs of menstrual MSC origin exhibited facilitatory and inhibitory effects, respectively, on cortical neural progenitor cells [92]. Differences in the contents between exosomes and MVs may be one of the reasons for the resulting opposing functions [33]. Furthermore, different types of stem cells and different conditions of the culture environment are also sources of heterogeneity. This heterogeneity poses difficulties for the commercialization of EV products due to the inability to guarantee consistent product quality. Finally, and most importantly, the therapeutic effects of EVs show a dose-dependent nature, which requires EVs to be produced in higher yields or isolated more efficiently while maintaining high purity. While the ISEV's position statement in the 2018 update has yet to endorse any isolation method capable of maintaining both high yields and high purity, new isolation techniques in recent years offer hope. Two new technologies, tangential flow filtration [93] and deep filtration [94], have been reported to simultaneously maintain product purity while increasing yield, improving the separation efficiency of target EVs. These novel technologies may become the mainstream methods of choice for EV treatment in the future. In addition, it may be possible to improve EV performance in vivo by appropriately modifying the culture medium composition or microenvironment or by applying genetically engineered modifications to the cells used for EV production. Through antitumour experiments, researchers induced MSC uptake of antitumour drugs by using high concentrations of paclitaxel in conditioned medium, and they found that these MSCs secreted EVs containing high levels of paclitaxel and exhibiting potent antitumour activity in vivo and in vitro [95]. Possible strategies for donor cell modification include genetic modification of source cells, direct loading of exogenous drugs, and artificial nanovesicle fusion [96]. Specifically, inserting target genes into donor cells or introducing plasmids containing target genes can increase the levels of target proteins or miRNAs within EVs [97]. Through direct coincubation, hydrophobic drugs bind to the EV lipid bilayer, although this passive loading strategy usually results in low loading levels [98]. Artificial liposomes are formed by encapsulating drug molecules in vesicles formed from phospholipid bilayers [99]. The shortcomings of artificial liposomes include low drug encapsulation rates, low preservation stability, and vulnerability to clearance by the immune system [100]. Stem cell-derived EVs can compensate for these disadvantages. The fusion of liposomes and EVs may be the basis of a new and promising drug delivery system [101]. The aforementioned modification strategies may hold promise for the development of EV therapy by improving efficacy.

## Conclusion

Overall, as a cell-free component, the unique properties of EVs such as their low immunogenicity and cytotoxicity, their high structural and compositional stability, their natural receptor cell and organ targeting ability, and their ability to freely pass complex biological barriers make EVs a promising next-generation therapeutic approach.

## Data Availability

Not applicable.

## Abbreviations

AAV: Adeno-associated virus
AAV2: Adeno-associated virus vector serotype 2
AMD: Age-related macular degeneration
Bax: Bcl-2-associated X protein
BBB: Blood–brain barrier
BCL-2: B cell lymphoma 2
BDNF: Brain-derived neurotrophic factor
BMSC-EVs: Bone marrow mesenchymal stem cell-derived extracellular vesicles
BMSCs: Bone marrow mesenchymal stem cells
BRB: Blood‒retinal barrier
Brn3a^+^: Brain specific homeobox/POU domain protein 3a-positive
CCL2: C–C motif chemokine ligand 2
CCL12: C–C motif chemokine ligand 12
CNS: Central nervous system
DR: Diabetic retinopathy
ESCRT: Endosomal sorting complexes required for transport
EVs: Extracellular vesicles
FTMHs: Full-thickness macular holes
GAP43^+^: Growth-associated protein 43-positive
GSH-Px: Glutathione peroxidase
hADSC: Human adipose mesenchymal stem cell
hESC: Human embryonic stem cell
HMGB1: High-mobility group box 1
HOXC6: Homeobox C6
hucMSC: Human umbilical cord MSC
ICAM-1: Intercellular adhesion molecule 1
IL: Interleukin
ILVs: Intraluminal vesicles
ISEV: International Society for Extracellular Vesicles
ITGA1: Integrin subunit α1
MHC I: Major histocompatibility class I
miRNA: Micro RNA
MNU: *N*-methyl-*N*-nitrosourea
MSC-EVs: Mesenchymal stem cell-derived extracellular vesicles
MSCs: Mesenchymal stem cells
mTOR: Mammalian target of rapamycin
MVBs: Multivesicular bodies
MVs: Microvesicles
ncRNAs: Noncoding RNAs
NF-κB: Nuclear factor kappa B
NLRP3: The nucleotide-binding domain and leucine-rich repeat-containing family, pyrin domain-containing 3
PDCD4: Programmed cell death 4
PI3K: Phosphoinositide 3-kinase
PRP: Platelet-rich plasma
PRRs: Pattern recognition receptors
PTEN: Phosphatase and tensin homolog
RGCs: Retinal ganglion cells
ROS: Reactive oxygen species
RPE: Retinal pigment epithelium
SNAREs: Soluble N-ethylmaleimide-sensitive factor attachment protein receptors
SOD: Superoxide dismutase
STAT1: Transcription 1
TGF-β: Transforming growth factor beta
Th1: T helper 1
Th17: T helper 17
TLR: Toll-like receptor
TNF-α: Tumour necrosis factor-α
VCAM-1: Vascular cell adhesion molecule 1
